# Comparison of Biostimulant Treatments in *Acmella oleracea* Cultivation for Alkylamides Production

**DOI:** 10.3390/plants9070818

**Published:** 2020-06-29

**Authors:** Stefania Sut, Irene Ferrarese, Shyam Sharan Shrestha, Gourav Kumar, Antonio Slaviero, Simone Sello, Adriano Altissimo, Luca Pagni, Francesco Gattesco, Stefano Dall’Acqua

**Affiliations:** 1 DAFNAE, Department of Agronomy, Food, Natural Resources, Animals and Environment, Agripolis Campus, University of Padova, 35020 Legnaro (PD), Italy; stefania.sut@studenti.unipd.it; 2 Department of Pharmaceutical and Pharmacological Sciences, University of Padova, Via Marzolo 5, 35131 Padova, Italy; Ire.ferrarese@gmail.com (I.F.); shyamsharan999@gmail.com (S.S.S.); gourav8490@gmail.com (G.K.); 3 Landlab Srl, 36050 Quinto Vicentino, Vicenza, Italy; antonioposta@yahoo.it (A.S.); s.sello@landlab.net (S.S.); a.altissimo@landlab.net (A.A.); 4 Indena SpA, 20090 Settala, Milano, Italy; luca.pagni@indena.com (L.P.); francesco.gattesco@indena.com (F.G.)

**Keywords:** *Acmella oleracea*, alkylammides, biostimulation, LC-MS, NMR, open field cultivation, triacontanol

## Abstract

*Acmella oleracea* is a promising cosmetic, nutraceutical, and pharmaceutical ingredient, and plants with high levels of active compounds are needed in the market. Cultivation can be valuable if sufficient levels of alkylamides are present in plant material. In this regard the application of biostimulants can be an innovative approach to increase yield of cultivation or bioactive compound levels. *A. oleracea* plants were cultivated in Northern Italy in an experimental site using three different types of biostimulants, triacontanol-based mixture (Tria), an extract from plant tissues (LL017), and seaweed extract (Swe). Plants were grown in the field in two different growing seasons (2018 and 2019). After treatments inflorescences were harvested and the quali-quantitative analysis of alkylamides and polyphenols was performed. Treated and control plants were compared for yields, morphometric measurements, quali-quantitative composition in secondary metabolites. Overall results show that both triacontanol-based mixture and the LL017 positively influenced plant growth (Tria >+ 22%; LL017 >+ 25%) and flower production (Tria >+ 34%; LL017 >+ 56%). The amount of alkylamides and polyphenols in flowers were between 2.0–5.2% and 0.03–0.50%, respectively. Biostimulant treatments ensure higher cultivation yields and allow maintenance of the alkylamide and polyphenol levels based on % (*w*/*w*), thus offering an advantage in the final quantity of extractable chemicals. Furthermore, data revealed that samples harvested in late season show a decrease of polyphenols.

## 1. Introduction

*Acmella oleracea* (L.) R. K. Jansen (commonly called jambu), Asteraceae, is a flowering Brazilian species largely diffused worldwide. It is grown as an ornamental or medicinal plant and it is also known as the “Toothache plant”. It is used as medicinal plant in different parts of the world in particular to treat oral pain because of its analgesic properties. When the leaves and flowers are chewed, they cause a tingling sensation to tongue and lips due to the presence of alkylamides, the most abundant in *A. oleracea* being spilanthol. This isobutylamide derivative presents local anesthetic action and this effect justifies the traditional use for treating toothache [1,2,3,4].

Jambu is attracting greater interest in the field of herbal medicine and nutraceuticals due to its potential analgesic, antioxidant, and anti-inflammatory activities, and several patent applications have been filed, showing the industrial potential of this plant for cosmetic and pharmaceutical applications [2,5]. 

In this regard, the cultivation of species for the use in cosmetic and pharmaceutical applications can be a challenge due to the need for standardized and high yield content of active constituents [6,7]. Different agronomic practices can be used with the aim to increase the secondary metabolites or the yield/ha of cultivated plants. The search for natural products by consumers is also related to more sustainable ways of production as well as to agricultural practices that limit the use of chemicals. 

There is increasing interest in sustainable ways of production, as well as in the agricultural practices that limit the use of chemicals, in food but also in the area of natural products for pharmaceutical, nutraceutical, and cosmetic uses.

Biostimulants are products that are applied to plants or soils to regulate and enhance the crop’s physiological processes, thus making them more efficient [8,9,10,11,12,13]. To our knowledge, limited information related to the use of biostimulants in the cultivation of medicinal and officinal species has been published so far. Many natural substances have been investigated as biostimulants in crops, and four types of constituents have been generally considered, namely triacontanol-containing products, plant-derived protein hydrolysates, seaweeds, and microorganisms [8,9,10,11,12,13,14]. In this study, a Triacontanol-based mixture (Tria), an extract from plant tissues (LL017), and a seaweed extract (Swe) were used at two different application doses (Low and High). Previous published research reported the triacontanol-mediated improvement of several parameters in various crops, such as growth, yield, photosynthesis, protein synthesis, uptake of water and nutrients, nitrogen-fixation, enzymatic activities and contents of free amino acids, reducing sugars, soluble proteins, and active constituents as essential oil. The application of plant-derived extracts stimulates plant uptake of macro- and micronutrients, and helps in rapid plant growth and biomass accumulation, interfering with carbon and nitrogen metabolic activities [8,9,10]. Seaweed extracts were largely used in the agricultural practice for their plant growth-promoting effects and for their ameliorating effect on crop tolerance to abiotic stresses such as salinity, extreme temperatures, nutrient deficiency, and drought [8,15]. 

The selected biostimulant treatments were considered in open field conditions to investigate the morphometric changes in plants grown during the hot season. Normal fertilization (UTC 100% fertilized) CTR1 and double fertilization (UTC 200% fertilized) CTR2 were used as control conditions.

The aim of the study was to investigate the morphological modification and the levels of bioactive compounds in *A. oleracea* plants cultivated treated with different biostimulants. Secondary metabolites content was chemically characterized with different analytical methods in *A. oleracea*. N-Alkylamides are considered to be the most significant class of metabolites of this plant, and mostly contain a poly-unsaturated aliphatic fatty acid chain and a shorter substituent at the amide side. Plant biomass and flower production were monitored in both control and biostimulant treated *A. oleracea* plants. Nuclear magnetic resonance (NMR) and liquid chromatography coupled with mass spectrometry (LC-DAD-MS^n^) were used as analytical approaches to assess chemical constituents, allowing the annotation and the quantification of different alkylamides and polyphenols studying their modification in treated and control groups. 

## 2. Results

### 2.1. Plant Growth and Flower Production

#### 2.1.1. Plant Growth in Season 2018

*A. oleracea* seeds were initially sown in pots in the greenhouse, and they were maintained in controlled conditions until plant dimensions were sufficient for their establishment and growth, by mid-June. 

**Figure 1:**
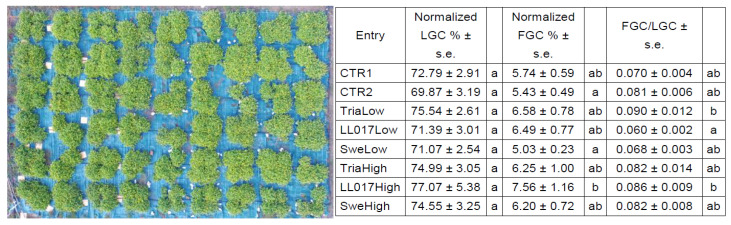
Plant dimension assessment on 08.10.18. Representative image of a drone picture (**left**) used for the determination of plant and flower quantification. Living ground cover (LGC), flower ground cover (FGC), and their ratio (**right**) resulting from the statistical analysis of numerical data obtained from the picture. The statistical analysis (software Statistica by StatSoft) was performed by means of one-way analysis of variance (one-way ANOVA) with Duncan Test (α) = 0.1. For each trait, at least one letter in common indicates no significant difference according to the Duncan test.

Since the homogeneity of plants was not similar in and among plots (see Figure 1 left), the final dimension of plants was assessed by the elaboration of drone pictures with WinCAM NDVI (Regents Instruments Inc.) to generate data that quantify the covering of plants (living ground cover—LGC) and flowers (flower ground cover—FGC) at the end of growing season (08.10.18). LGC and FGC values were normalized on the number of plants present in every plot and data analyzed (Figure 1 right). 

Results indicate that the double nutrition did not affect plant dimensions and the LGC variation among entries did not exceed 10%. On the other hand, FGC was more variable among entries, with a maximum difference of about 50%. This makes FGC more relevant than LGC, as flowers are the plant part used for metabolites extraction. In these terms, TriaLow results in giving the highest FGC/LGC ratio compared to all other entries. Moreover, data suggest a dose-response effect, as TriaHigh showed a lower ratio than TriaLow (0.082 vs. 0.090), but differences are not statistically significant compared to CTR1. The only statistically significant difference in terms of FGC/LGC is between LL017Low (0.060 ± 0.002) and TriaLow (0.090 ± 0.012) and LL017High (0.086 ± 0.009).

#### 2.1.2. Plant Growth in Season 2019

*A. oleracea* was sown again in January 2019 in pots in the greenhouse and transplanted in May since plants used in the previous year did not survive the winter. Since they were more homogeneous than 2018, their growth was followed by the measurement of plant height and diameter on 12.08.19, 02.09.19, and 24.09.19. No statistically significant differences were recorded on the first date, after plants received two treatments. The second assessment was carried out after four treatments and the last after plants were treated eight times. Results indicate that treated entries gradually differentiated compared to the untreated control, and the same was observed for CTR2. In particular, on the last day of assessment, LL017Low resulted to be the condition that gave the tallest plants +34.7% than CTR1, and LL017High gave larger plant diameters than all other entries (+32.8% than CTR1) (Table 1). These differences are statistically significant compared to CTR1, their untreated control. In addition, all treated entries gave statistically larger plant diameters than CTR1 at the last date of assessment (24.09.19). The general trend for treated entries is that products fill the gap between CTR1, the single fertilized entry and CTR2, the double fertilized one, in terms of plant diameter. In fact, values from treated entries are very close to what was observed for CTR2 (Table S1 for the analysis of CTR1 vs. treated entries).

### 2.2. Flower Production in 2019 

Flowers were harvested two times during the season, in September and October. Inflorescences were weighed per plot and data analyzed. The entry that produced the total highest quantity of material was TriaLow, with 962.00 ± 148.84 g in total (+84.54% than CTR1). All the other treated entries gave lower yield than CTR2, but still more than CTR1, in detail Tria High +34.01%, SweHigh +40.74%, LL017Low +56.82%, SweLow +59.16%, LL017High +61.75% (Table 2).

Taken together, flower production results indicate that a trend can be detected in both growing seasons, with highest values recorded for TriaLow and LL017High, with CTR2 that also scored good results.

### 2.3. NMR Analysis of Acmella

**Figure 2:**
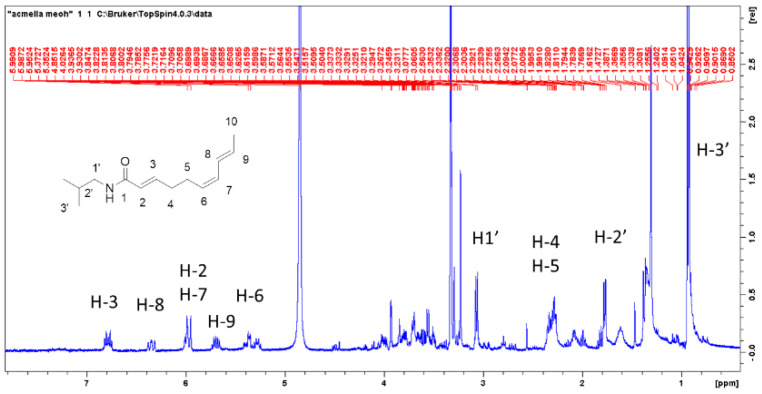
^1^H NMR of methanolic extract of *Acmella oleracea* (commercial sample)**.**

**Figure 3:**
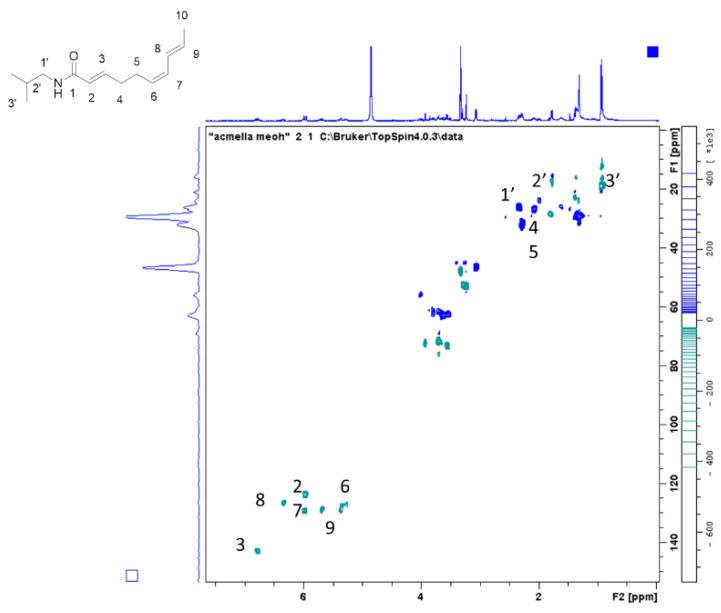
Spilanthol assignment in the HSQC-DEPT spectrum in extract obtained from reference plant material.

*A. oleracea* is known for containing spilanthol but the compound is not commercially available as standard. For this reason, from reference plant material methanol extract was prepared and screened by the use of ^1^H-NMR due to the ability of the technique to detect all kinds of phytoconstituents and due to the fact that any compound presents the same response factor. Two commercial plant materials of *A. oleracea* were extracted with methanol and used for NMR analysis. As can be seen in the ^1^H-NMR spectrum reported in Figure 2, signals due to the presence of spilanthol are easily detectable, and in agreement with literature data [16]. This result indicates a significant amount of compound in the extract. 2D spectra namely HSQC-DEPT (Figure 3), HMBC, COSY, and TOCSY were recorded to confirm the assignment of the main compound and to have information on minor constituents. Confirmation of the structure of the main alkylamide in these samples was obtained due to long range H-C correlations in HMBC and due to homonuclear coupling signals revealed by COSY spectrum. HMBC spectrum allowed to assign also quaternary positions as carbonyl function at C1 and COSY coupling allowed to confirm the relative position of double bonds in the chain. Minor signals can be ascribed to other constituents as sugar, while no significant signals related to phenolic constituents can be detected at this concentration suggesting that the polyphenol amount in the plant material is significantly lower than alkylamide.

**Figure 4:**
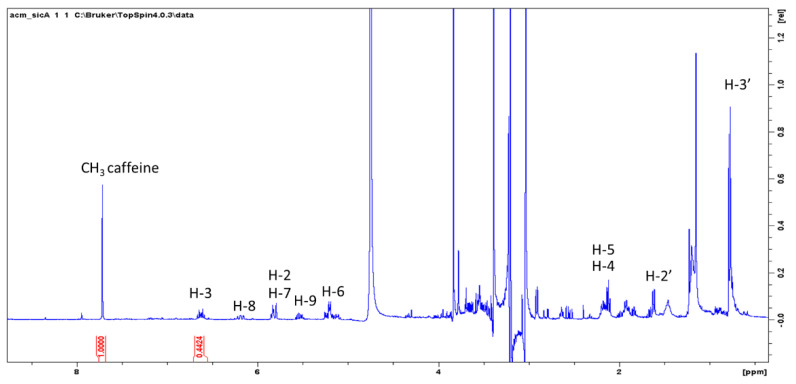
^1^H NMR of in commercial sample of *A. oleracea* with internal standard caffeine.

Considering the high abundance of spilanthol in methanol extract of *A. oleracea* and the need for reference compound for spilanthol, total akylamides quantification was performed by NMR with the use of caffeine as internal standard (Figure 4) using an approach that we previously applied to other plant extracts [17,18,19]. Once the amount of spilanthol in commercial extracts was determined, these were then used as reference for the quantification in cultivated samples using the LC approach. These results indicate the possible use of direct ^1^H-NMR analysis for the determination of alkylamides in *A. oleracea* with a fast and economic method. Total alkylamides in REF1 (*Acmella oleracea* “Sicilia” C/213365 28/02/18) and REF2 (*Acmella oleracea* C/211016 28/02/2018) were 1.45 ± 0.05% and 0.926 ± 0.002%, respectively.

### 2.4. LC-MS Chemical Characterization

**Figure 5:**
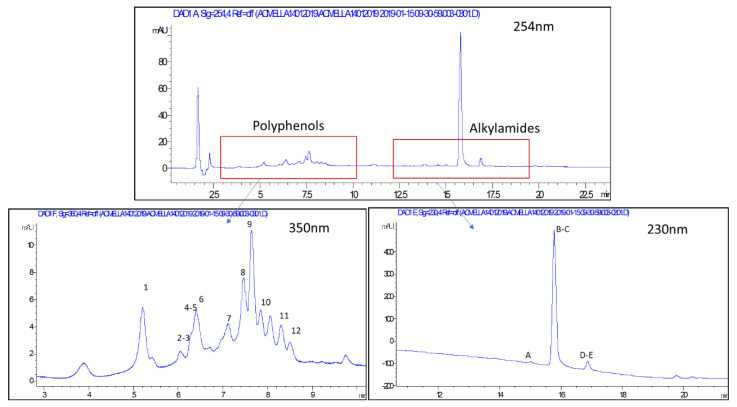
Chromatogram of *A. oleracea* extract at 254 nm and enlargements at 350 nm of polyphenol signals and at 230 nm of alkylamide signals.

Different papers report quantification methods by GC or GC-MS for the analysis of alkylamides of *A. oleracea* [16,20,21], and polyphenols were analyzed in another paper by HPLC-DAD-ESI/MS^n^ [22]. With the aim to investigate the chemical fingerprint of samples, we decided to use LC-DAD-MS^n^ approach. LC-MS allows to observe different classes of phytoconstituents coupling the resolution of chromatographic approach and the selectivity and sensitivity of MS-based methods. Diode array detector (DAD) was also used in parallel to MS detection to have a complete view of *A. oleracea* phytoconstituents. LC traces are reported, showing the chromatograms recorded with the DAD highlighting the main classes of compounds (Figure 5).

**Figure 6:**
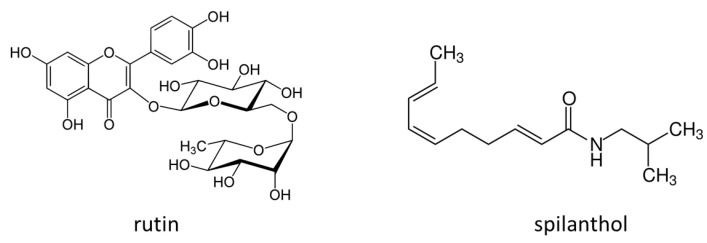
Chemical structure of rutin and spilanthol.

Qualitative results are reported in Table 3. The tentatively assigned constituents, their retention times, [M-H]^−^ or [M+H]^+^ and fragments are reported. The polyphenols fingerprint in *A. oleracea* presents several peaks showing a UV spectrum that supports the presence of quercetin and kaempferol derivatives (max at 350, 290 nm), furthermore a peak presenting a UV spectrum typical of caffeic acid derivatives was observed (max 330 nm). MS^n^ analysis revealed that the polyphenols are mainly quercetin, kaempferol, and hydroxycinnamic derivatives (detected in negative ion mode), while alkylamides were observed in positive ion mode. Polyphenols reported in Table 3 are in agreement with previously compounds reported for *A. oleracea* extract [22] except for the ions at *m/z* 593 (tr 6.1) and *m/z* 651 (tr 7.7). In a previous paper only O glycosylated quercetin derivatives were tentatively identified, and in our samples, we annotated a C glycosylated quercetin and a kaempferol derivative. These variations can be related to area of cultivation or to different climatic conditions. On the other hand, literature report the presence of cyanidin derivatives [22], but in our analysis these compounds were not searched. For alkylamides, all the annotated ions were previously reported and our analysis confirmed the presence of spilanthol as main derivative and a similar pattern of other minor constituents as reported in the literature [1,20,21]. For our purposes, the LC-DAD-MS^n^ method appeared to be the most feasible because it allows the contemporary analysis of both polyphenols and alkylamides. Quantitative analysis revealed limited changes in the relative amount of each annotated compound in the analyzed samples. Due to the aim of this paper, that was to assess the biochemical effects within the plants, the data of polyphenols and alkylamides contents were reported as sum of total polyphenol derivatives and total alkylamide derivatives. In Figure 6 the structure of rutin and spilanthol was reported.

#### 2.4.1. Alkylamides Content 

Results obtained from different harvests (11.09.2018, 11.10.2018 first year, 19.09.2019, 21.10.2019 second year) are reported in Table 4.

The samples from open field-cultivated plants present high values of total alkylamides compared with reference samples obtained in other cultivation sites in Southern Italy and used as reference (average spilanthol amount 0.92–1.45%). Alkylamide amount in this trial ranged between 2.28% and 5.76%. In 2018, the average content of alkylamide was 3.08% for the first harvest and 4.76% for the second harvest, showing an increase of 1.5-fold. In 2019 the first and second harvest average values were similar, namely 5.09% and 5.06%.

The amount of alkylamides was affected by biostimulant treatments only in a few cases and differences between 2018 and 2019 were observed.

The average value measured from CTR1 samples in the first harvest of 2018 was 3.4%. Only the treatments with TriaLow and SweHigh showed variation compared to control, being lower than the two CTR (*p* < 0.05). Considering CTR2, the amount of alkylamide was increased for treatment SweLow and LL017High (*p* < 0.1).

The amount of alkylamide was increased for treatment SweLow compared to CTR2 (*p* < 0.1) in the second harvest in 2018. On the other hand, comparison of all the treatments with CTR1 yielded a lower average amount. Comparing the two harvests of 2018, the SweLow appears to be the most promising treatment.

Considering data collected in the two years in this open field trial, no biostimulant treatments cause changes in alkylamide content (% *w*/*w*) in *Acmella* canopy.

#### 2.4.2. Polyphenols Content

Results related to polyphenol contents are summarized in Table 5. In order to observe potential effects of the biostimulant on *A. oleracea* plants, these secondary metabolites were also considered although their amount in this species is significantly lower compared to alkylamides.

The polyphenols amount was 10 times less compared to alkylamides, indicating that the most abundant class of secondary metabolites was the latter one, confirming the ^1^H-NMR data. The average amount of total polyphenols in *A. oleracea* flowers was 0.41% and 0.35% for first and second harvest in 2018, and 0.08% and 0.05% for first and second harvest of 2019, showing a four-fold decrease between 2018 and 2019.

TriaLow (*p* < 0.1), LL017Low (*p* < 0.05), and TriaHigh (*p* < 0.05) treatments increased the content of polyphenols compared to CTR2 in the first harvest of 2018. Limited variation among all treatments in terms of polyphenols content (*p* > 0.1) was observed for the flowers of the second harvest in 2018. The average polyphenols amount from samples CTR1, CTR2, and LL017 Low was higher compared to the other treatments (*p* > 0.1) in the first harvest of 2019. The average amount of polyphenols decreased for all treatments compared to the first harvest, and all concentrations ranged between 0.04% and 0.06% in the second harvest of 2019.

Overall, the amount of polyphenols in the first harvest was higher for all the entries compared to the second harvest, showing a decreased polyphenols content with the beginning of autumn both in 2018 and 2019 samples. 

## 3. Discussion

Biostimulants aim to increase plant production and/or their quality by improving their fitness. Their mode of action is not well understood yet, but much evidence supports the beneficial effects of biostimulation. These aspects are of interest especially if plants considered are used for cosmetic, nutraceutical, and pharmaceutical applications.

Our results on the application of six different biostimulant treatments on *A. oleracea* plants showed a good impact on plant growth and production, with a limited effect on the biosynthesis of secondary metabolites.

Triacontanol-based mixture (Tria), plant tissues extract (LL017), and seaweed extract (Swe) at all dosages improved plant growth, especially in 2019, when treated plants nearly reached the entry with double nutrition in terms of dimensions at the end of the season. This result is significant, as plant growth positively affects flower production, an energy-consuming process. Our results indicate that all treated entries produced a higher flower biomass compared to the untreated control (CTR1), with TriaLow that reached +84.54%. Moreover, biostimulants filled the gap between single and double fed plants in terms of flower production. Regarding 2018 plant traits, the plants were very inhomogeneous and data were not used. Nevertheless, LL017High and TriaLow gave the highest indexes of flowers on total canopy.

Regarding the biosynthesis of bioactive compounds, the obtained results indicate limited influence of biostimulant treatments on the % (*w*/*w*) of alkylamides and polyphenols. Higher levels of alkylamides were observed in the field trial compared to other reference standard plant material that was cultivated in different geographic areas. For *A. oleracea* in the experimental conditions, the considered biostimulant treatments resulted in a content of secondary metabolites that was not significantly improved considering their % amount (*w*/*w*) at harvest times compared to control plants. Results indicate additional information since the open field trials gave higher metabolites content than plants cultivated in pots in greenhouse conditions (Tables S3 and S4). This might be due to a much lower level of environmental stimuli in the greenhouse, that maintained plants in less stressed conditions, with a subsequently lower level of secondary metabolites synthesis. 

*A. oleracea* flowers are the plant part that is more required for alkylamides extraction, and for this reason, on the basis of our results, we suggest that TriaLow is the best treatment to be carried out to increase the production of this plant material. Moreover, results indicate that triacontanol fills the gap between normal and over-fertilized plants, since TriaLow produced more flowers than CTR2, the double fertilized entry. Overall results, combining morphological traits and chemical data, indicate that the quantity of alkylamides is very similar among treated and non-treated plants, and a higher biomass production induced by biostimulants is very desirable in terms of final yield for extractable metabolites.

For the both years the last harvest of *A. oleracea* showed decreased levels of polyphenols, suggesting a strong influence of the harvest time.

## 4. Materials and Methods 

### 4.1. Open Field Experimental Site and Plant Cultivation

*A. oleracea* plants were cultivated in 2018 and 2019 in Landlab research station (Quinto Vicentino-VI-Italy, 45.57° N, 11.62° E, 33 masl) in an open field with native soil substrate, sandy-loam, pH 7–7.5. Seeds were sown on peat to provide plants that were transplanted in the field once established. Mulching on plots was provided in order to avoid the overlap of plants from different plots. Once transplanted at the beginning of the summer, plants were trimmed in order to make the canopy uniform. Solid fertilizer (ILSA Professional NPK (8-6-14) solid granules at 160 kg/ha (double NPK) or 80 kg/ha (base NPK)) was applied three times to satisfy the nutrient demand of plants, that was estimated in 80 kg/ha N, 60 kg/ha P, 140 kg/ha K for 100% fertilized and 160 kg/ha N, 120 kg/ha P, and 280 kg/ha K for 200% fertilized. Plants were cultivated in randomized complete block plots, with six replicates per entry and four plants per replicate. Irrigation was provided by means of plant-by-plant drip system in order to maintain moisture in the substrate.

### 4.2. Biostimulant Treatments

Three different types of biostimulant were prepared and used on the basis of literature search. Triacontanol-based biostimulant obtained by combining enzymatic hydrolysate from Fabaceae species with supercritical extract rich in triacontanol, an extraction from plant tissues, and a seaweed-based product (Acadian Seaplants, Canada).

The triacontanol-based biostimulant was prepared using a mixture of a plant hydrolysate and a combination of concentrated solution of triacontanol obtained by extraction from the same species. Two different products were prepared, one containing 6.5 ppm of triacontanol and one containing 100 ppm of triacontanol with a previously validated method [24]. Briefly, for liquid products 1000 mg of material were weighed, the internal standard (IS) solution was added (1000 uL of a 500 ug/mL solution), and extracted in a flask with 50 mL of dichloromethane. Extraction was performed in ultrasound bath for 15 min. An Agilent 1100 HPLC system (Agilent Technologies, Santa Clara, CA, USA) coupled to a Sedere Sedex 60 ELSD detector (Olivet, France) was used. In order to elute highly lipophilic compounds from the reverse phase column used as stationary phase (Agilent Extend C-18 4.6 × 150 mm, 5 µm), a gradient of acetonitrile (A) and methanol/methyl tertbutyl ether 10/90 (B) was used as mobile phase. Gradient conditions were optimized in order to perform the analysis in 30 min and to reach the best separation of TRIA and the IS. The gradient started with 10% B, in 15 min it went to 40% B, then isocratic up to 26 min. Flow was 1 mL/min, injection was 10 µL.

The aminoacidic analysis in triacontanol based biostimulant was performed by HPLC-MS/MS as we previously described [25]. Briefly, samples were extracted in an ultrasound bath with a solution of diluted HCl (0.5 M) for 10 min at room temperature. Standard solutions were prepared, weighing the exact amount of each amino acid in diluted HCl solution (0.5 M) in four different concentrations from 1 to 10 ug/mL. For hydrolysis, samples were added of 15% trichloroacetic acid (10 mL) and left at 80 °C for one night in sealed vials. pH of solution was then adjusted with ammonia solution (33%) to 2.0 and solutions were used for analysis. For analysis, an Agilent Z-HILIC Column was used as stationary phase (3 × 100 mm, 4 micron), eluents were acetonitrile, (A) and water 0.5% formic acid (B). The gradient started with 1 min to 95% A, then in 11 min to 70% A, then 14 min to 40% A, then at 14.5 min back to 95% A. Flow rate was 0.450 mL/min. Each amino acid transition was optimized with corresponding standard solution. The amino acid profile of triacontanol based biostimulant was reported in Table 6.

The plant extract contained 8.9 ppm of triacontanol and an amino acid profile reported in Table 7.

Seaweed biostimulant composition was analyzed for polysaccharides and content was 10% (*w*/*w*). Products were also analyzed for the content of gibberellic, gibberellinic, indol-3-acetic acid, and none of the products contained these constituents at the detection limit (<5 ug/mL).

Entries considered in the trials were:

1. UTC 100% fertilized; CTR1
2. UTC 200% fertilized; CTR2
3. 100% fertilized + 6.5 ppm Triacontanol UniPd 3 L/ha; TriaLow
4. 100% fertilized + LL017 3 L/ha; LL017Low
5. 100% fertilized + Acadian SWE 1.5 L/ha; SweLow
6. 100% fertilized + 100 ppm Triacontanol UniPd 3 L/ha; TriaHigh
7. 100% fertilized + LL017 50 L/ha; LL017High
8. 100% fertilized + Acadian SWE 15 L/ha; SweHigh

Plants were treated once per week starting from the full establishment of the plants, until the last harvest, for a total of 15 times in 2018 and 13 times in 2019.

Products were applied with foliar application using a hand sprayer on well-developed plants at 3 L/ha for 6.5 (low) or 100 (high) ppm Triacontanol, 3 (low) or 50 (high) L/ha LL017, and 1.5 (low) or 15 (high) L/ha Acadian SWE in 500 L/ha of water as carrier.

Monitoring of weather conditions: local weather conditions were monitored by using the weather station Vantage pro2 (Davis Instruments Corp. Inc., CA), located in Landlab (45.57° N, 11.62° E, 33 masl), that is 20 m from the trial location.

### 4.3. Biometric Traits of the Plants in Open Field

Living ground cover (LGC) and flower ground cover (FGC) for plants grown in 2018 in open field were assessed from pictures taken by a drone (Phantom 3 Advanced, DJI, China).

The image was analyzed using WinCAM NDVI (Regents Instruments Inc., CA) to generate data that quantified the covering of plants and flowers. Data were normalized on the number of plants per plot in case of the death of some plants.

Biometrics of plants grown in the field in 2019 were assessed in terms of height, diameter, and flower yield by using a meter and a scale, respectively. Assessment were carried out after one, four, and eight treatments. 

### 4.4. Acmella oleracea Harvest

Fully developed inflorescences were manually harvested every four treatments, once a month. Samples from each entry were maintained separately and they were dried at 40 °C in the oven for 48 h. 

### 4.5. NMR Analysis

NMR analysis was used to assess the general phytochemical contents of the samples. Dried plant material (500 mg) (sample C/213365 28/02/18) was extracted with methanol, the solvent was removed under vacuum and dissolved in 700 microliter of deuterated methanol and used for the analysis.

### 4.6. NMR Quantification of Alkylamides

Preliminary investigation on the possibility to quantify spilanthol by ^1^H-NMR were performed using caffeine as internal standard as previously reported for other plant constituents [17,18,19]. The extraction was performed weighing 100 mg of dried plant material, adding 1 mg of caffeine, and extracting in methanol in ultrasound bath for 10 min with 25 mL of methanol. Liquid was decanted and collected, a further 20 mL of methanol was added, and the mixture was sonicated for 10 min. Liquids were collected, dried under vacuum, and the residue dissolved with deuterated methanol. Alternatively, 100 mg of plant material can be directly extracted with methanol and internal standard (1–3 mg) and the mixture used for ^1^H-NMR determination using solvent suppression.

An example of this determination is reported. Imidazole signal of caffeine can be used as reference while H-3 of spilanthol can be used for the measurement.

### 4.7. Qualitative Analysis of Acmella oleracea by LC-DAD-MS^n^

A total of 25 mg of *Acmella oleracea* powder was extracted with 15 mL of methanol, sonicated for 10 min, and supernatant was filtered. A second extraction was performed with another 10 mL of methanol, sonicated for 10 min. Solution was pulled with a final volume of 25 mL. Solution was used for LC analysis.

LC-DAD-MS^n^ allowed to obtain detailed fingerprinting of the extract due to the typical UV spectra of separated compounds and due to specific information obtained by the MS^n^ approach. After preliminary trials due to the presence of compounds that are easily ionized in negative ion mode in the first part of the chromatogram and due to the preferable analysis of alkylamides in positive ion mode the spectra were acquired up to 15 min in negative and after 15 min in positive ion mode. 

The instrumentation was Agilent 1260 chromatograph (Santa Clara, CA, USA) equipped with 1260 diode array detector (DAD) and Varian MS-500 ion trap mass spectrometer equipped with ESI source. At the end of the column, two “T” splitters separated the flow rate: half of the liquid was split to DAD and half to Agilent/Varian MS-500 ion trap mass spectrometer. UV-Vis spectra were acquired in the range of 190–400 nm. The LC separation was obtained on an Agilent C-18 XDB 3.0 × 150 mm (3 micron), as mobile phases acetonitrile (A) and water 0.1% formic acid (B). Flow rate was 0.4 mL/min. Gradient starts with 10% A, then 75% A in 15 min, then 10 min isocratic, then 90% A in 1 min. The MS parameters were different in negative mode (from 0 to 15 min) and in positive mode (from 15 to 31 min), in order to improve the ionization of polyphenol ions and alkylamide derivatives, respectively. The parameters in negative mode were as follows: capillary voltage 85 volt, RF loading 100%; nebulizer gas pressure, 25 psi; drying gas pressure, 25 psi; drying gas temperature, 265 °C; needle voltage, ±5000 V; spray shield voltage, 600 V. Mass spectra was acquired in negative mode in the spectral range 50–1500 Da. The parameters in positive mode were as follows: capillary voltage 85 volt, RF loading 85%; nebulizer gas pressure, 25 psi; drying gas pressure, 25 psi; drying gas temperature, 265 °C; needle voltage, ±4000 V; spray shield voltage, 600 V. Mass spectra were acquired in negative mode in the spectral range 50–900 Da. Data obtained was used to tentatively assign an identification to secondary metabolite compound on the base of literature references in the MS^n^ pathway.

For polyphenol Acmella samples were quantified with DAD detector. Rutin was used as standard compound for flavonoids, while chlorogenic acid was used as standard compounds for caffeic acid derivatives, and stock solutions were prepared at concentration of 60 ug/mL in methanol. Then dilutions were prepared in methanol in the range between 6 and 60 ug/mL and the calibration curve was as follows, y = 32.5x + 0.3 (R^2^ = 0.993) for rutin, y = 17x + 0.5 (R^2^ = 0.993) for chlorogenic acid. Peaks with UV spectra with maximum at 350 nm, ascribable to flavonoid structure, were selected and area was obtained at 350 nm. Peaks with UV spectra with maximum at 330 nm, ascribable to caffeoyl derivatives, were selected and the area was obtained at 330 nm. Quantitative data of the various samples were reported as sum of all the different quantified constituents.

For alkylamides, quantification was performed with a DAD detector. Since spilanthol was not available as reference standard, standardized commercial extract of Acmella was quantified by NMR. This extract was then used as reference for the quantification in cultivated samples using LC approach. Standardized extract was extracted with the same procedure of cultivated samples and serial dilutions were performed in methanol in the range of 5–50 ug/mL. Calibration of spilantol was as follows: y = 73,575x + 20.152 (R^2^ = 0.993). Peaks with UV spectra with maximum at 230 nm, ascribable to alkylamide structure, were selected and area was obtained at 230 nm.

## 5. Conclusions

This trial showed the possibility of using biostimulants for the cultivation of *Acmella oleracea* to increase the yield of production of material containing significant alkylamide levels. *A. oleracea* samples were analyzed with different techniques, namely NMR and LC-DAD-MS^n^, to investigate phytochemical composition. The main compound present in samples was spilanthol but other alkylamide derivatives and polyphenols were found in minor amounts. Biostimulant treated *A. oleracea* plants showed alkylamide and polyphenol amounts comparable with control plants (as % of dried weight). Nevertheless, a substantial increase of the biomass production of both vegetative parts and inflorescences was observed for plants treated with plant-based and seaweed-based biostimulants in *Acmella oleracea* cultivated in an open field in Northern Italy.

Overall, the obtained results present an example of an explorative study for the improvement of innovative agronomic approaches in the cultivation of officinal and medicinal plants using biostimulants.

## Figures and Tables

**Table 1:** Plant dimension assessment. Plant height and diameter were assessed three times during the 2019 growing season. Average values per plot were used for the analysis. The percentage increase for the last assessment (24.09.19) relative to the CTR1 is reported on the right of the respective column. Four and five outlier values were removed for height and diameter, respectively, by using the boxplot function (software Statistica by StatSoft) (coefficient = 1.5) before averaging plot values. The statistical analysis (software Statistica by StatSoft) was performed by means of one-way analysis of variance (one-way ANOVA) with Duncan Test (α) = 0.1. For each trait, at least one letter in common indicates no significant difference according to the Duncan test.

Entry Name	Plant Height (cm) ± s.e. 12.08.19		Plant Height (cm) ± s.e. 02.09.19		Plant Height (cm) ± s.e. 24.09.19		%	Plant Diameter (cm) ± s.e. 12.08.19		Plant Diameter (cm) ± s.e. 02.09.19		Plant Diameter (cm) ± s.e. 24.09.19		%
CTR1	7.06 ± 0.47	a	9.88 ± 1.33	a	13.44 ± 1.86	a	100.0	13.00 ± 1.83	a	19.69 ± 4.00	a	36.19 ± 5.54	a	100.0
CTR2	7.65 ± 0.68	a	12.67 ± 0.81	ab	16.82 ± 1.34	ab	125.1	17.15 ± 2.87	a	28.35 ± 3.06	b	47.85 ± 2.42	b	132.2
TriaLow	7.90 ± 0.31	a	12.40 ± 0.83	ab	16.60 ± 1.29	ab	123.5	16.78 ± 2.02	a	26.65 ± 2.53	b	47.32 ± 3.77	b	130.8
TriaHigh	8.03 ± 0.72	a	12.28 ± 1.14	ab	16.40 ± 1.51	ab	122.0	19.50 ± 3.26	a	29.13 ± 2.69	b	47.55 ± 3.49	b	131.4
SweLow	8.25 ± 0.76	a	12.85 ± 0.67	b	17.25 ± 1.05	ab	128.3	17.17 ± 1.23	a	27.75 ± 1.92	b	47.62 ± 2.16	b	131.6
SweHigh	7.10 ± 0.48	a	11.90 ± 0.47	ab	16.40 ± 0.77	ab	122.0	14.80 ± 1.02	a	26.10 ± 1.36	ab	46.55 ± 1.65	b	128.6
LL017Low	8.45 ± 0.84	a	13.35 ± 1.58	b	18.10 ± 1.58	b	134.7	18.80 ± 3.32	a	28.00 ± 2.71	b	45.40 ± 2.01	b	125.5
LL017High	7.95 ± 0.83	a	13.10 ± 1.25	b	17.20 ± 1.79	ab	128.0	18.05 ± 2.08	a	27.65 ± 3.08	b	48.05 ± 3.68	b	132.8

**Table 2:** Fresh biomass of harvested inflorescences. Flowers were manually harvested on 19.09.19 and 21.10.19. The percentage increase of the total biomass relative to CTR1 is reported on the right of the column of total yield. Three outlier values were removed by using the boxplot function (software Statistica by StatSoft) (coefficient = 1.5). The statistical analysis (software Statistica by StatSoft) was performed by means of one-way analysis of variance (one-way ANOVA) with Duncan Test (α) = 0.1. For each trait, at least one letter in common indicates no significant difference according to the Duncan test.

Flower Biomass (g) ± s.e.							
Entry Name	19.09.19		21.10.19		Total		%
CTR1	121.40 ± 35.13	a	399.90 ± 83.85	a	521.30 ± 114.04	a	100.00
CTR2	280.55 ± 43.67	a	647.61 ± 54.81	ab	928.16 ± 90.66	b	178.05
TriaLow	243.55 ± 48.43	a	718.45 ± 106.31	b	962.00 ± 148.84	b	184.54
LL017Low	240.88 ± 59.59	a	576.64 ± 75.18	ab	817.52 ± 96.18	ab	156.82
SweLow	217.88 ± 31.91	a	611.80 ± 70.05	ab	829.68 ± 99.59	ab	159.16
TriaHigh	181.50 ± 65.41	a	517.10 ± 77.02	a	698.60 ± 137.68	ab	134.01
LL017High	244.60 ± 71.05	a	598.60 ± 75.70	ab	843.20 ± 131.12	ab	161.75
SweHigh	170.24 ± 31.30	a	563.44 ± 37.05	ab	733.68 ± 57.25	ab	140.74

**Table 3:** Tentatively assigned secondary metabolites by LC-MS^n^ in *A. oleracea* extracts, annotation of compounds was obtained comparing spectral data with literature and reference when available. Reference available for alkylamides identification [1,3,21] and for polyphenols [22,23]. * comparison with reference standard.

n	rt	[M − H]^−^	Fragments	Tentatively Identified Polyphenol
1	5.4	771	625 505 446 301 271	Quercetin-3-O-sophoroside-7-O-rhamnoside
2	6.1	771	625 609 591 505 301 271	Quercetin-3-O(2-O-hexosyl,3-O-rhamnosyl) hexoside
3	6.1	593	503 473 383 353 297	Quercetin-6,8-di-C-hexoside
4	6.3	755	609 447 343 301 271	Quercetin-3-O-(rhamnosyl-2-O-glucosyl)-7-O-rhamnoside
5	6.3	609	429 343 301 271	Quercetin-O-hexosyl-O-rhamnoside
6	6.4	625	505 445 301 271	Quercetin-3-O-sophoroside
7	7.1	609	301 271	Rutin *
8	7.5	651	609 505 447 301	Quercetin- acetyl- O-hexoside- O- rhamnoside
9	7.7	651	609 591 471 285 255	Kaempferol-acetyl-3-O-sophoroside
10	7.9	667	625 505 445 301	Quercetin-O-acetyl-sophoroside
11	8.3	505	463 301	Querceti-acetylhexoside
12	8.5	515	353 175	3,5-di-caffeoylquinic acid
n	rt	[M + H]^+^	fragments	Tentatively identified Alkylamides
A	15.1	246	125	2E-N-(2-methylbutyl)-2,6,8-decatrienamide
B	15.8	222	125 84 70	spilanthol
C	15.9	232	177 107	2E-N-Isobutyl-2-undecene-8,10-diynamide
D	16.8	258	125	2E, 7Z-N-isobutyl-2,7-tridecadiene-10,12-diynamide
E	16.8	236	125 84	2E,6Z,8E-N-(2-methylbutyl-2,4,8-decatirenamide

**Table 4:** Alkylamide contents from different harvests (11.09.2018, 11.10.2018 first year, 19.09.2019, 21.10.2019 second year) for each treatment. Values expressed are means ± S.D. of three parallel measurements for plant material samples. # *p* ≤ 0.05 vs. CTR1 (11.09.2018) * *p* ≤ 0.05 vs. CTR2 (11.09.2018) ◦ *p* ≤ 0.1 vs. CTR2 (11.09.2018) $ *p* ≤ 0.05 vs. CTR1 (11.10.2018) • *p* ≤ 0.1 vs. CTR1 (11.10.2018) α *p* ≤ 0.1 vs. CTR2 (11.10.2018).

	11.09.2018	11.10.2018	19.09.2019	21.10.2019
Samples	% Alkylamides			
CTR1	3.40 ± 0.31	5.31 ± 0.10	4.78 ± 0.20	5.34 ± 1.16
CTR2	3.17 ± 0.09	4.67 ± 0.03 $	5.12 ± 0.80	5.00 ± 0.61
TriaLow	2.27 ± 0.13 #*	4.90 ± 0.22 •	5.76 ± 0.76	5.01 ± 1.00
LL017Low	3.10 ± 0.19	4.56 ± 0.12 $	5.45 ± 0.67	5.04 ± 0.46
SweLow	3.40 ± 0.08 ◦	4.91 ± 0.17 •α	4.65 ± 1.32	5.01 ± 0.48
TriaHigh	3.23 ± 0.13	4.54 ± 0.38 $	4.92 ± 0.51	4.78 ± 0.57
LL017High	3.39 ± 0.12 ◦	4.54 ± 0.17 $	5.25 ± 0.65	4.68 ± 1.00
SweHigh	2.70 ± 0.09 #*	4.64 ± 0.35 $	4.79 ± 0.59	5.59 ± 1.28
mean	3.08	4.76	5.09	5.06

**Table 5:** Polyphenol contents from different harvests (11.09.2018, 11.10.2018 first year, 19.09.2019, 21.10.2019 second year) for each treatment. Values expressed are means ± S.D. of three parallel measurements for plant material samples. # *p* ≤ 0.05 vs. CTR2 (11.09.2018) * *p* ≤ 0.1 vs. CTR2 (11.09.2018).

	11.09.2018	11.10.2018	19.09.2019	21.10.2019
Samples	% Polyphenols			
CTR1	0.36 ± 0.06	0.36 ± 0.04	0.12 ± 0.08	0.06 ± 0.03
CTR2	0.37 ± 0.05	0.34 ± 0.02	0.10 ± 0.11	0.05 ± 0.03
TriaLow	0.40 ± 0.07 *	0.37 ± 0.03	0.08 ± 0.06	0.06 ± 0.04
LL017Low	0.43 ± 0.03 #	0.32 ± 0.04	0.10 ± 0.10	0.04 ± 0.02
SweLow	0.41 ± 0.04	0.36 ± 0.05	0.07 ± 0.03	0.06 ± 0.04
TriaHigh	0.48 ± 0.04 #	0.35 ± 0.04	0.08 ± 0.05	0.05 ± 0.03
LL017High	0.39 ± 0.05	0.35 ± 0.04	0.07 ± 0.05	0.05 ± 0.04
SweHigh	0.41 ± 0.05	0.35 ± 0.03	0.07 ± 0.06	0.06 ± 0.04
mean	0.41	0.35	0.08	0.05

**Table 6:** The aminoacidic analysis in triacontanol based biostimulant. Values are reported as %m/m.

		Free	Total
Aspartic acid	%	0.071 ± 0.02	0.2655 ± 0.02
Glutamic acid	%	0.078 ± 0.02	0.409 ± 0.03
Alanine	%	0.244 ± 0.05	0.381 ± 0.01
Arginine	%	0.007 ± 0.002	0.0468 ± 0.01
Phenylalanine	%	0.100 ± 0.02	0.034 ± 0.01
Glycine	%	0.188 ± 0.02	0.594 ± 0.03
Hydroxyproline	%	0.078 ± 0.01	0.266 ± 0.01
Isoleucine	%	0.034 ± 0.01	0.109 ± 0.04
Histidine	%	0.006 ± 0.01	0.754 ± 0.01
Leucine	%	0.063 ± 0.005	0.135 ± 0.01
Lysine	%	0.0406 ± 0.020	0.144 ± 0.01
Methionine	%	0.003 ± 0.001	0.0441 ± 0.01
Proline	%	0.137 ± 0.02	0.484 ± 0.01
Serine	%	0.015 ± 0.001	0.100 ± 0.01
Tyrosine	%	0	0
Threonine	%	0	0.0157 ± 0.01
Valine	%	0.109 ± 0.02	0.153 ± 0.01
Asparagine	%	0.077 ± 0.01	0.094 ± 0.01
Cysteine and Cystine	%	0.046±0.01	0.071 ± 0.01
Total amount		1.3	4.1

**Table 7:** The aminoacidic analysis in plant extract biostimulant. Values are reported as %m/m.

		Free	Total
Aspartic acid	%	0.083 ± 0.01	0.343 ± 0.02
Glutamic acid	%	0.090 ± 0.01	0.529 ± 0.04
Alanine	%	0.281 ± 0.01	0.492 ± 0.03
Arginine	%	0.007 ± 0.001	0.061 ± 0.01
Phenylalanine	%	0.115 ± 0.02	0.044 ± 0.02
Glycine	%	0.217 ± 0.02	0.767 ± 0.02
Hydroxyproline	%	0.0902 ± 0.01	0.343 ± 0.04
Isoleucine	%	0.039 ± 0.01	0.141 ± 0.02
Histidine	%	0.0072 ± 0.001	0.973 ± 0.01
Leucine	%	0.072 ± 0.01	0.174 ± 0.01
Lysine	%	0.046 ± 0.01	0.187 ± 0.02
Methionine	%	0.003 ± 0.001	0.056 ± 0.01
Proline	%	0.157 ± 0.01	0.626 ± 0.03
Serine	%	0.0180 ± 0.01	0.130 ± 0.01
Tyrosine	%	0	0
Threonine	%	0	0.020 ± 0.01
Valine	%	0.126 ± 0.01	0.198 ± 0.01
Asparagine	%	0.088 ± 0.01	0.121 ± 0.03
Cysteine and Cystine	%	0.054 ± 0.01	0.093 ± 0.01
Total amount		1.5	5.3

## Supplementary Materials

The following are available online at https://www.mdpi.com/2223-7747/9/7/818/s1, weather conditions, Figure S1: Rain, maximum, average, and minimum temperatures of the field trial, Table S1: Differences in plant height and diameter between CTR1 (base fertilized entry) and the treated entries, Table S2: Differences in the final biomass of harvested inflorescences between CTR1 (base fertilized entry) and the treated entries, Table S3: Alkylamide content in plants grown in pots in the greenhouse in 2018. Table S4: Polyphenols content in plants grown in pots in the greenhouse in 2018.

## Abbreviations

CTR1	UTC 100% fertilized
CTR2	UTC 200% fertilized
TriaLow	100% fertilized + 6.5 ppm Triacontanol UniPd 3 L/ha
LL017Low	100% fertilized + LL017 3 L/ha
SweLow	100% fertilized + Acadian SWE 1.5 L/ha
TriaHigh	100% fertilized + 100 ppm Triacontanol UniPd 3 L/ha
LL017High	100% fertilized + LL017 50 L/ha
SweHigh	100% fertilized + Acadian SWE 15 L/ha
FGC	flower ground cover
LGC	living ground cover
