# Supplementary material for: Comparison of Biostimulant Treatments in Acmella oleracea Cultivation for Alkylamides Production
Source: Plants (Basel). 2020 Jun 29;9(7):818. doi: 10.3390/plants9070818 (PMC7411836; doi:10.3390/plants9070818)
Supplement: Supplementary file 1 [file plants-09-00818-s001.pdf]

Supplementary Figures:

Weather conditions

Weather conditions were recorded throughout the whole period of *A. oleracea* cultivation in open air, and data are reported from May to October 2018–2019. These periods range from the transplant to the last harvest, and rain, maximum, average, and minimum temperatures are reported. The general trend for 2018 shows quite high temperatures especially in May, with a warmer spring than in 2019. Then, from June to October, the situation was almost the same for both years. The only difference is that 2018 was slightly rainier than 2019. A detailed description for weather conditions in 2019 is reported below.

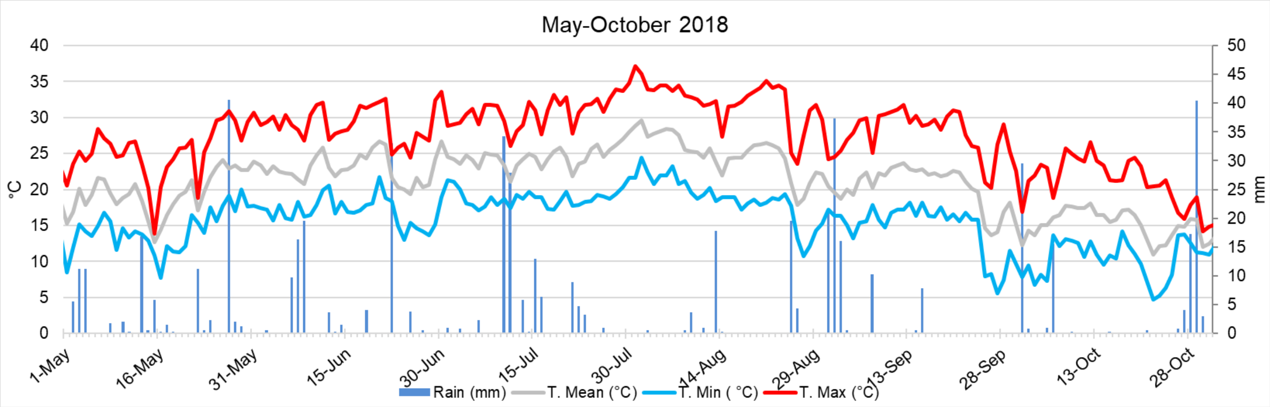

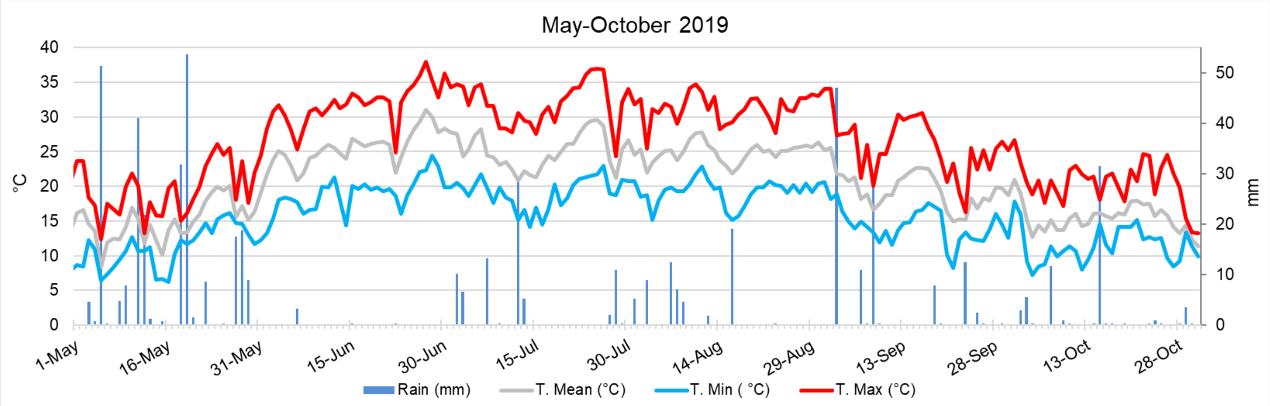

Figure S1. Rain, maximum, average, and minimum temperatures of the field trial.

Weather conditions in 2019 were very far from the climatological average values recorded in 2018 since the beginning of Spring 2019, with noticeable variations in short time periods.

The daily mean temperatures in May ranged from 15 to 20 °C, almost always below the average (20 °C). Daily solar irradiance was 4.4 kWh/m^2, almost 40% less than the daily irradiance expected on sunny days.

In June there was a dramatic change for both temperatures and solar energy. Daily temperature average rose to 25.5 °C, nearly 4.5 °C more than the climatological average (21 °C). In June only two days (06.06.19 and 22.06.19) showed temperatures aligned with the climatological average. Solar irradiance was close to its maximum almost every day, with ca. 6.3 kWh/m^2. This means that the increase of daily energy per unit of surface from the end of May to the beginning of June was of 30%.

In July weather conditions were slightly less severe than in June, with a mean daily temperature of 25.1 °C (1.6 °C more than the climatological average) and mean daily solar irradiance of ca. 5.8 kWh/m^2.

In August weather conditions were much hotter than the average for most of the month, which had a mean temperature of 24.9 °C and 24 days with temperatures above 30 °C. The solar radiation

was almost always close to its maximum (5.8 kWh/m^2). Only three rain events were recorded, with a total rainfall of 54 mm. With the only exception of the 2nd August, characterized by a temporary fresh phase, full summer conditions were continuously experienced all month.

A sudden decrease in temperatures was recorded in September starting from the 2nd day of the month. The monthly average temperature was 19.8 °C with six events of rain with a total rainfall of 111 mm.

October was 2 °C hotter than the average and very dry, while November recorded 24 rainy days and almost 250 mm of monthly rainfall.

Supplementary Tables:

Table S1. Differences in plant height (above) and diameter (below) between CTR1 (base fertilized entry) and the treated entries.

**Plant diameter (cm) ± s.e.**									
Condition	12.08.19		% increase	02.09.19		% increase	24.09.19		% increase
CTR1	13.00 ± 1.23	a		19.69 ± 4.00	a		36.19 ± 5.54	a	
others	17.52 ± 0.48	b	34.74	27.55 ± 0.23	b	39.92	47.08 ± 0.57	b	30.10
Plant height (cm) ± s.e.									
Condition	12.08.19		% increase	02.09.19		% increase	24.09.19		% increase
CTR1	7.06 ± 0.47	a		9.88 ± 1.33	a		13.44 ± 1.86	a	
others	7.94 ± 0.12	b	12.53	12.65 ± 0.16	b	28.07	16.99 ± 0.24	b	26.45

Data from treated entries were grouped to obtain the same number of data from CTR1, they were analyzed by means of one-way analysis of variance (one-way ANOVA) with Duncan Test (α) = 0.1. For each trait, at least one letter in common indicates no significant difference according to the Duncan test. Results indicate that biostimulant treatments significantly improve plant dimensions up to nearly 40%.

Table S2. Differences in the final biomass of harvested inflorescences between CTR1 (base fertilized entry) and the treated entries.

**Flower biomass (g) ± s.e.**									
Condition	19.09.19		% increase	21.10.19		% increase	Total		% increase
CTR1	121.40 ± 35.13	a		399.90 ± 83.85	a		521.30 ± 114.04	a	
others	216.79 ± 4.09	b	78.57	597.83 ± 16.75	b	49.49	814.62 ± 18.78	b	56.27

Data from treated entries were grouped to obtain the same number of data from CTR1, they were analyzed by means of one-way analysis of variance (one-way ANOVA) with Duncan Test (α) = 0.1. For each trait, at least one letter in common indicates no significant difference according to the Duncan test. Results indicate that biostimulant treatments significantly improve flower biomass to a total of 56.27%.

Table S3. Alkylamide content in plants grown in pots in the greenhouse in 2018.

	**02/10/2018**	**12/11/2018**	**21/12/2018**
Samples		% Alkylamide	
CTR1	2.88 ± 0.60	3.82 ± 0.27	2.49 ± 0.12
CTR2	2.58 ± 0.31	3.79 ± 0.27	3.74 ± 0.31
TriaLow	2.33 ± 0.34	4.06 ± 0.25	3.12 ± 0.15
LL017Low	3.18 ± 0.85	3.82 ± 0.22	2.60 ± 0.09
SweLow	2.50 ± 0.23	4.08 ± 0.18	3.02 ± 0.02
TriaHigh	2.31 ± 0.42	4.50 ± 0.13	2.58 ± 0.95
LL017High	2.21 ± 0.59	3.61 ± 0.32	2.11 ± 0.14
SweHigh	2.20 ± 0.35	4.24 ± 0.06	3.15 ± 0.21
Mean	2.52	3.99	2.85

Table S4. Polyphenols content in plants grown in pots in the greenhouse in 2018.

	**02/10/2018**	**12/11/2018**	**21/12/2018**
Samples		% Polyphenols	
CTR1	0.31 ± 0.03	0.42 ± 0.01	0.20 ± 0.05

CTR2	0.24 ± 0.04	0.20 ± 0.04	0.22 ± 0.04
TriaLow	0.17 ± 0.03	0.34 ± 0.01	0.22 ± 0.02
LL017Low	0.21 ± 0.01	0.29 ± 0.03	0.25 ± 0.01
SweLow	0.26 ± 0.02	0.31 ± 0.02	0.23 ± 0.03
TriaHigh	0.35 ± 0.03	0.35 ± 0.02	0.23 ± 0.07
LL017High	0.26 ± 0.02	0.27 ± 0.04	0.20 ± 0.01
SweHigh	0.25 ± 0.02	0.37 ± 0.02	0.19 ± 0.05
Mean	0.26	0.32	0.22
